# The trends of disease burden due to high temperature in Mainland China from 1990 to 2019 and its prediction to 2030

**DOI:** 10.1038/s41598-023-49491-6

**Published:** 2023-12-14

**Authors:** Jiaolong Zheng, Haiyan Lin, Jingyi Ling, Jiaofeng Huang, Dongliang Li

**Affiliations:** 1 Department of Hepatobiliary Disease, The 900th Hospital of Joint Logistics Support Force, No. 156 Northern Xi’er Huan Road, Fuzhou, 350025 China; 2 Department of Hepatobiliary Disease, Fuzong Clinical Medical College of Fujian Medical University, Fuzhou, China; 3 Department of Hepatology, Hepatology Research Institute, The First Affiliated Hospital of Fujian Medical University, Fuzhou, 350025 China

**Keywords:** Attribution, Risk factors

## Abstract

The risk of high-temperature-related diseases is increasing owing to global warming. This study aimed to assess the trend of disease burden caused by high temperatures in Mainland China from 1990 to 2019 and to predict the trend of disease burden over the next 10 years. The latest data were downloaded from the Global Burden of Disease Database (GBD) for analysis, and the disease burden related to high temperature was described by mortality and disability-adjusted life-years (DALYs) and stratified by etiology, sex, and age. Statistical analyses were performed using the R software. In 2019, there were 13,907 deaths attributed to high temperatures in Mainland China, and this was 29.55% higher than the 10,735 deaths in 1990. Overall, the age-standardized mortality and DALYs attributed to high temperatures showed a downward trend from 1990 to 2019. We observed an etiological shift in high-temperature-related diseases. The age-standardized DALYs contribution attributed to high temperatures in 1990 was mainly from communicable, maternal, neonatal, and nutritional diseases (CMNND) (21.81/100,000), followed by injury (18.30/100,000) and non-communicable diseases (10.40/100,000). In 2019, the largest contribution shifted to non-communicable diseases (10.07/100,000), followed by injuries (5.21/100,000), and CMNND (2.30/100,000). The disease burden attributed to high temperatures was higher in males than in females and increased with age. In 2030, the mortality rate and DALYs due to high temperatures are predicted to decrease further, and the largest contribution will come from chronic non-communicable diseases, the occurrence of which will remain at a high level over the next 10 years. The burden of disease due to high temperatures in Mainland China is still heavy, mainly due to population aging and an increase in non-communicable diseases.

## Introduction

Global warming is indisputable. Intergovernmental Panel on Climate Change reports clearly show that the global average surface temperature increased by 0.85 °C from 1880 to 2012^1^. Global climate change has a huge impact on humans, which may not only lead to environmental changes in biological species but also cause changes in the disease spectrum^2–4^. Published studies suggest that the morbidity and mortality rates of many diseases are increasing with global warming^5,6^. Kendra et al. found that high temperatures are closely related to an increase in emergency department visits and hospitalization rates for cardiovascular diseases^7^. The results showed that direct heat illness morbidity and mortality increased by 18% for every 1 °C increase in temperature^8^. Scholars have also found that for every 1 °C increase in temperature, the risk ratio for respiratory disease mortality is 1.063 and that for cardiovascular disease mortality is 1.046^9^. In addition, high temperatures can increase the mortality rate of enteric infections and stroke^10,11^.

The Global Burden of Disease (GBD) is a systematic tool that contains data on diseases, injuries, and risk factors from various regions worldwide and provides burden data for 369 diseases and injuries and 87 risk factors^11,12^. GBD data are widely used by governments to inform health policies, resource allocation, and disease prevention and control^13^. Song et al. found that the disease burden attributable to high temperatures varied in different countries and showed an upward trend in high-temperature-related non-communicable diseases^14^. An estimate of the disease burden caused by high temperatures in Mainland China remains unknown. In this study, we aimed to investigate the trends in disease burden due to high temperatures in Mainland China from 1990 to 2019 and make predictions for 2030 according to the latest data from the GBD.

## Methods

### Study data

Data were obtained from the 2019 GBD of Disease database (https://vizhub.healthdata.org/gbd-results/). The GBD dataset was hosted by the Institute for Health Metrics and Evaluation at Washington University. GBD data come from various national censuses, household interviews, medical security records, disease registration, and environmental monitoring. The collected data were evaluated and transformed using the Bayesian meta-regression tool DisMod-MR 2.1 to estimate the mortality and disability-adjusted life years (DALYs) of disease burden over time^15^.

### Data screening

Deaths and DALYs were used as metrics to quantify the burden of high temperatures. The following selection criteria were applied in this study: analyses were stratified by age and sex. Age was categorized into five groups: 0–9 years old, 10–24 years old, 25–49 years old, 50–69 years old, and ≥ 70 years. The timespan for data retrieval was 1990–2019.

The causes of the high temperatures in the GBD were classified into four levels^12^.

According to the first level, the causes were mainly divided into three types: (1) communicable, maternal, neonatal, and nutritional diseases (CMNND); (2) non-communicable diseases, including cardiovascular diseases, chronic respiratory diseases, diabetes, and kidney diseases; and (3) injuries, including transport injuries, unintentional injuries, self-harm, and interpersonal violence.

### Definition

#### Age standardized rate (ASR)

The age-standardized rate (ASR) was used to estimate trends. The calculation of the ASR is based on the age structure of a certain population, which can reflect the constantly changing population composition patterns. In this study, the disease burden due to high temperatures was represented by the ASR of deaths and the ASR of DALYs.

#### Disability adjusted life years (DALYs)

DALYs refer to comprehensive indicators for calculating the sum of years of life lost and years of life lived with a disability and are widely used in disease burden research.

#### Estimated annual percentage change (EAPC)

The estimated annual percent change (EAPC) can be used as an indicator to evaluate the average annual percentage change in ASR over the selected period. The calculation formula is as follows: EAPC = 100 × (exp (exp(β) − 1)^16^. β is the regression coefficient in a linear regression model. If both EAPC and 95% CI did not contain 0, the disease showed an increasing or decreasing trend; otherwise, the disease showed a stable trend.

#### Prediction analysis

For the prediction of high temperatures burden, Nordpred model was used. Nordpred model was shown to have a relatively lower error rate before^17^. The average trend based on all observed data was extrapolated out to the year 2030^18^. Firstly, download the 1990–2019 demographic data and high-temperature related ASR from the GBD 2019 database, and read the predicted demographic data in the database (https://ghdx.healthdata.org/data-type/estimate). Subsequently, the R package Nordpred was used to predict the trend of burden due to high temperatures in the future.

### Statistical analysis

The data on death, DALYs, ASR, and 95% uncertainty interval (UI) due to high temperatures were collected. R software was used to extract and analyze the data. Trends in disease burden due to high temperatures were plotted using R software, and the sample was stratified by sex and age. The R package Nordpred was used to predict the trend in disease burden due to high temperatures over the next 10 years.

## Results

### Disease burden due to high temperature

**Figure 1:**
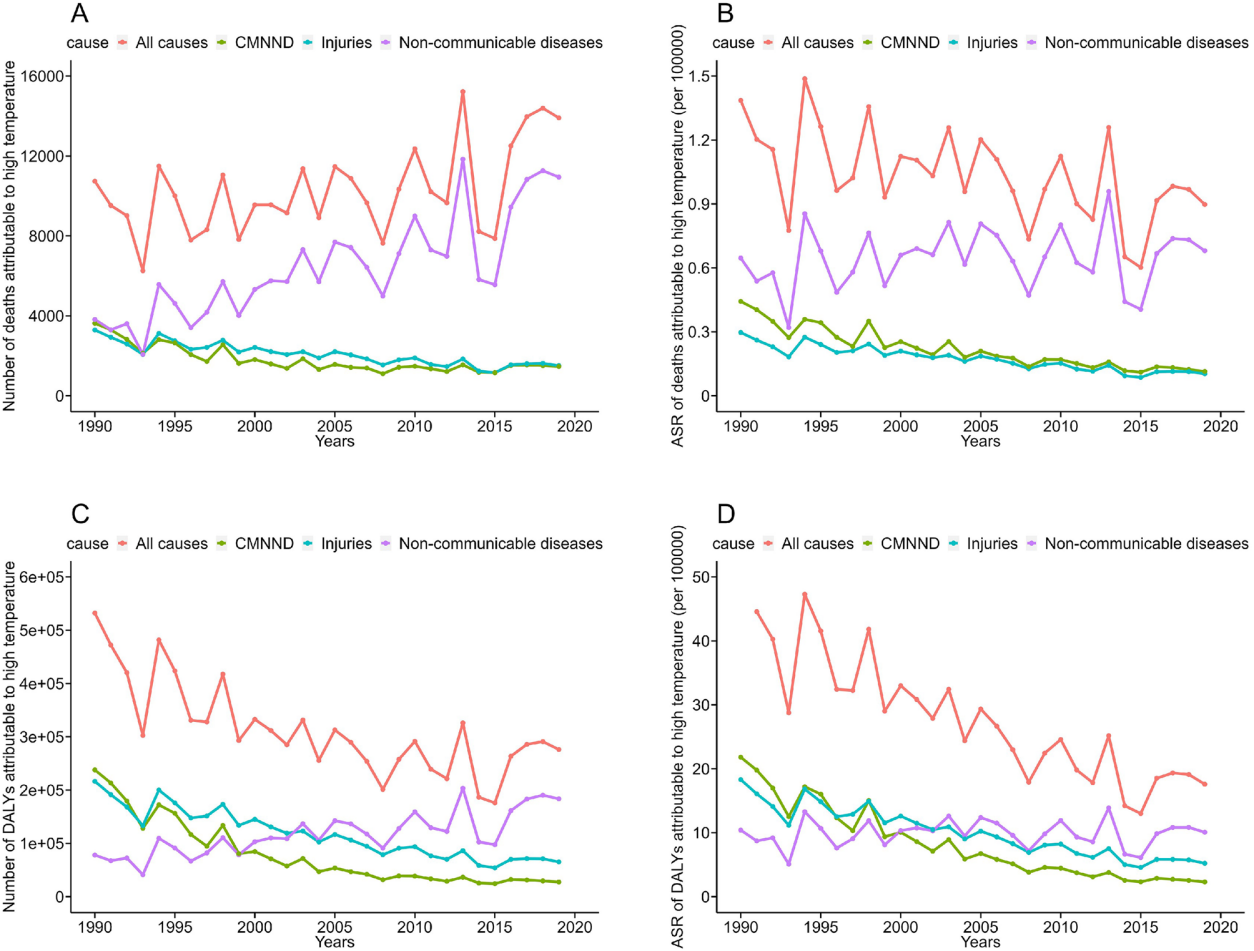
Trend of disease burden due to high temperatures from 1990 to 2019. (**A**) Number of deaths; (**B**) ASR of deaths; (**C**) number of DALYs; (**D**) ASR of DALYs.

The trends in the disease burden due to high temperatures in Mainland China are shown in Table 1. The deaths due to high temperatures increased by 29.55% between 1990 and 2019, from 10,735 cases to 13,907 cases. Death cases due to high temperatures showed a wave-like increasing tendency every year from 1990 to 2019 in Mainland China (Fig. 1A). The ASR of deaths showed a downward trend from 1990 (1.39/100,000) to 2019 (0.9/100,000), with an EAPC of − 1.26 (95% CI − 2.01 to − 0.49) (see Fig. 1B for details). In 2019, the cases and ASR of DALYs due to high temperature were 276,132 cases and 17.58/100,000, respectively, indicating a significant decrease compared to 1990 (EAPC =  − 3.56, 95% CI − 4.20 to − 2.91). (Fig. 1C,D) The causes of high temperatures were further divided into CMNND, non-communicable diseases, and injuries. The number and ASR of deaths due to high temperatures mainly originated from non-communicable diseases, followed by CMNND and injuries. The ASR of DALYs due to high temperatures in 1990 mainly came from CMNND (21.81/100,000), followed by injury (18.30/100,000) and non-communicable diseases (10.40/100,000). In 2019, there was a significant change in the ASR of DALYs attributed to high temperatures, with the largest contribution shifting to non-communicable diseases (10.07/100,000), followed by injuries (5.21/100,000) and CMNND (2.30/100,000).

**Table 1:** Trend of disease burden due to high temperature in Mainland China from 1990 to 2019.

Groups	1990		2019		EAPC in ASR (1990–2019, 95% CI)
	Number	ASR (per 100,000)	Number	ASR (per 100,000)	
Death					
Total	10,735	1.39	13,907	0.90	− 1.26 (− 2.01, − 0.49)
Cause					
CMNND	3626	0.44	1463	0.11	− 4.29 (− 4.8, − 3.77)
Non-communicable diseases	3818	0.65	10,940	0.68	0.44 (− 0.54, 1.43)
Injuries	3291	0.30	1504	0.10	− 3.44 (− 3.94, − 2.95)
Gender					
Male	5725	1.54	7850	1.23	− 0.35 (− 1.10, 0.40)
Female	5010	1.27	6057	0.69	− 1.99 (− 2.76, − 1.21)
Age					
0–9	3773	0.80	246	0.07	− 8.08 (− 8.63, − 7.53)
10–24	831	0.23	208	0.09	− 3.35 (− 3.91, − 2.80)
25–49	907	0.22	845	0.15	− 1.06 (− 1.67, − 0.45)
50–69	1772	1.15	2794	0.76	− 1.23 (− 2.03, − 0.42)
70+	3451	9.02	9814	9.09	0.37 (− 0.49, 1.22)
DALYs					
Total	532,219	50.52	276,132	17.58	− 3.56 (− 4.20, − 2.91)
Cause					
CMNND	237,881	21.81	27,441	2.30	− 7.65 (− 8.18, − 7.1)
Non-communicable diseases	78,001	10.40	183,593	10.07	0.13 (− 0.83, 1.09)
Injuries	216,336	18.30	65,098	5.21	− 4.19 (− 4.67, − 3.7)
Gender					
Male	302,146	55.73	174,213	23.73	− 2.72 (− 3.36, − 2.09)
Female	230,074	45.42	101,919	12.19	− 4.61 (− 5.28, − 3.93)
Age					
0–9	327,467	69.03	21,069	6.34	− 8.12 (− 8.67, − 7.57)
10–24	59,626	16.47	14,737	6.47	− 3.43 (− 3.99, − 2.86)
25–49	46,787	11.43	41,783	7.41	− 1.28 (− 1.89, − 0.67)
50–69	50,554	32.82	78,961	21.41	− 1.22 (− 2.02, − 0.42)
70+	47,785	124.90	119,581	110.76	− 0.13 (− 0.99, 0.74)

*CMNND* communicable, maternal, neonatal, and nutritional diseases.

### Stratified analysis by gender and age

**Figure 2:**
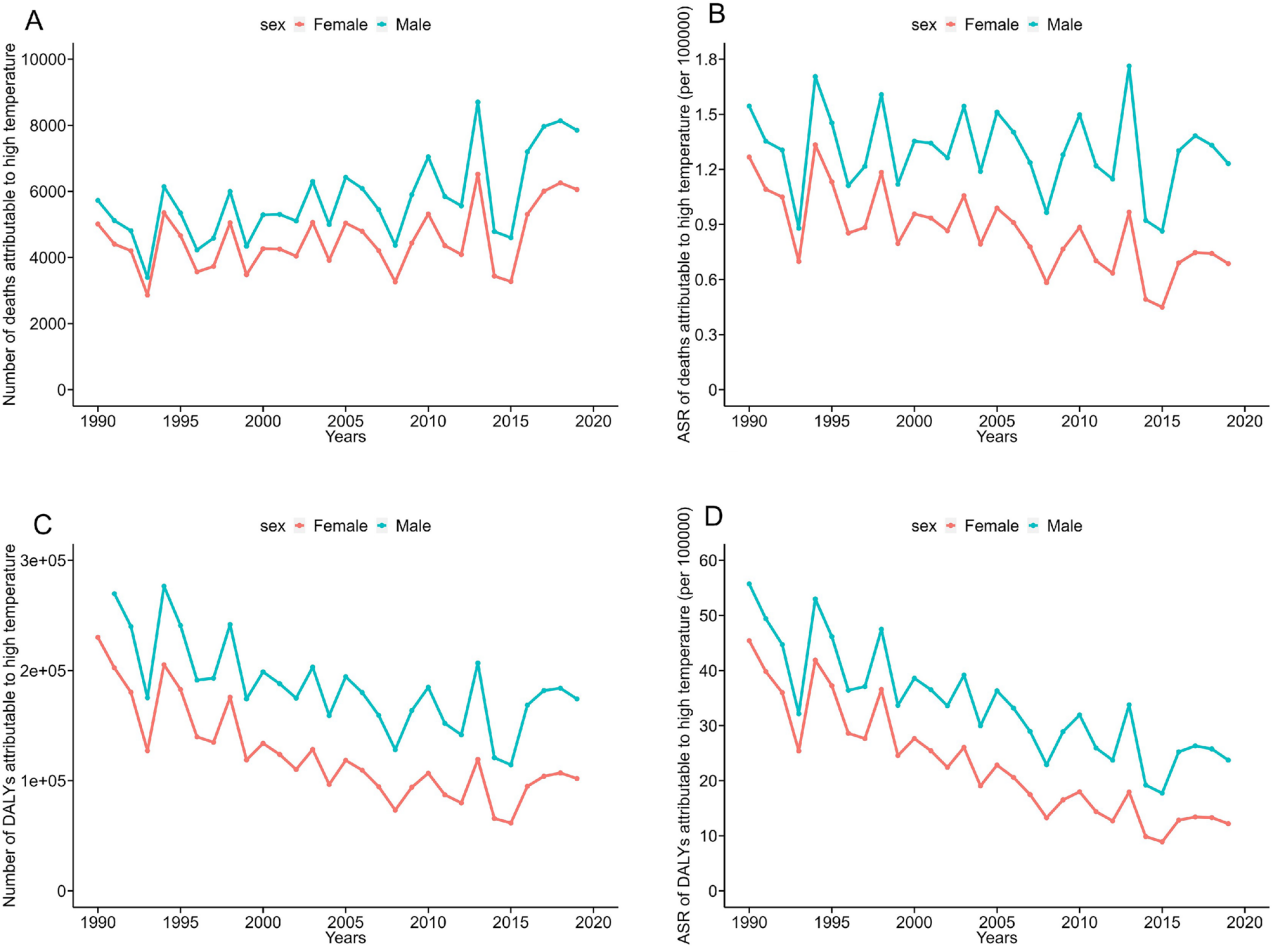
Trend of disease burden due to high temperatures from 1990 to 2019 grouped by gender. (**A**) Number of deaths; (**B**) ASR of deaths; (**C**) number of DALYs; (**D**) ASR of DALYs.

In 2019, the number and ASR of deaths were 7850 and 1.23/100,000 for males, respectively, which were far higher than those for females (6057 and 0.69/100,000, respectively). From 1990 to 2019, the ASR of deaths due to high temperatures in males showed a stable trend (95% CI of EAPC including 0), while in females it showed a decreasing trend (both EAPC and 95% CI were less than 0). In 2019, the DALYs and ASR of DALYs were 174,213 and 23.73/100,000 for males, respectively, which were significantly higher than those for females (101,919 and 12.19/100,000), respectively. From 1990 to 2019, the ASR of DALYs for both males and females showed a downward trend (Fig. 2A–D for details).

**Figure 3:**
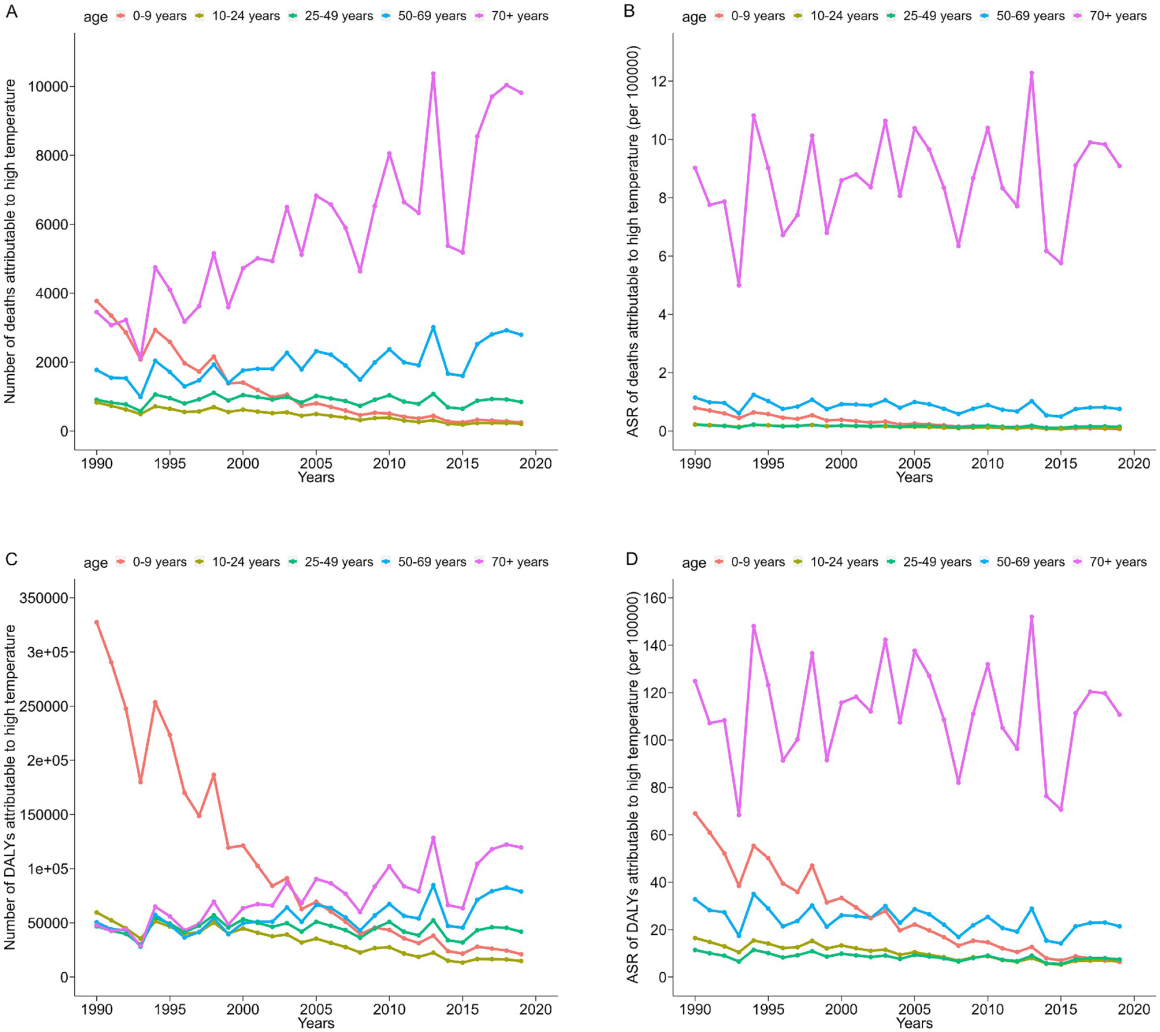
Trend of disease burden due to high temperatures from 1990 to 2019 grouped by age. (**A**) Number of deaths; (**B**) ASR of deaths; (**C**) number of DALYs; (**D**) ASR of DALYs.

The disease burden due to high temperatures varied significantly among the different age groups, as shown in Fig. 3A–D. The highest number of deaths in 2019 was in the ≥ 70 year-old-group (9814 cases), followed by those aged 50–69 (2794 cases) and 25–49 (845 cases). Overall, the number of deaths increases with age. The ASR of deaths also showed the same trend, with the highest in the ≥ 70 age group (9.09/100,000), followed by those aged 50–69 (0.76/100,000) and 25–49 (0.15/100,000). After further analysis of the trends in ASR of deaths among different age groups, it was found that the ≥ 70 year-old group showed a stable trend from 1990 to 2019 (95% CI of EAPC including 0), while other age groups showed a decreasing trend (EAPC < 0). In 2019, the heaviest burden on the ASR of DALYs was in the ≥ 70 age group, followed by the 50–69 and 25–49 age groups. Overall, the ASR of the DALYs due to high temperatures increased with age.

### Prediction analysis

**Figure 4:**
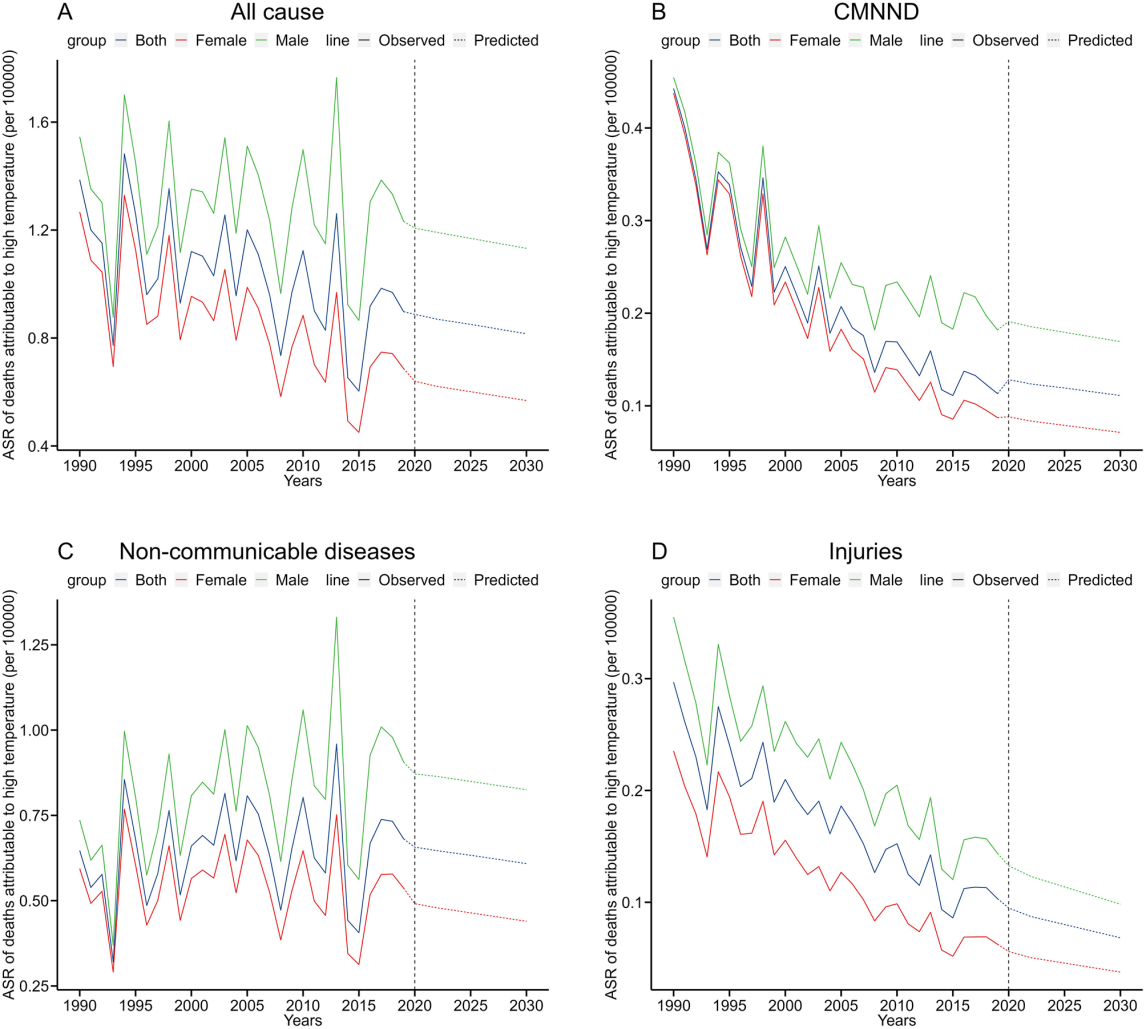
Prediction of mortality due to high temperatures in Mainland China from 2020 to 2030. (**A**) All cause; (**B**) CMNND; (**C**) non-communicable diseases; (**D**) injuries.

**Figure 5:**
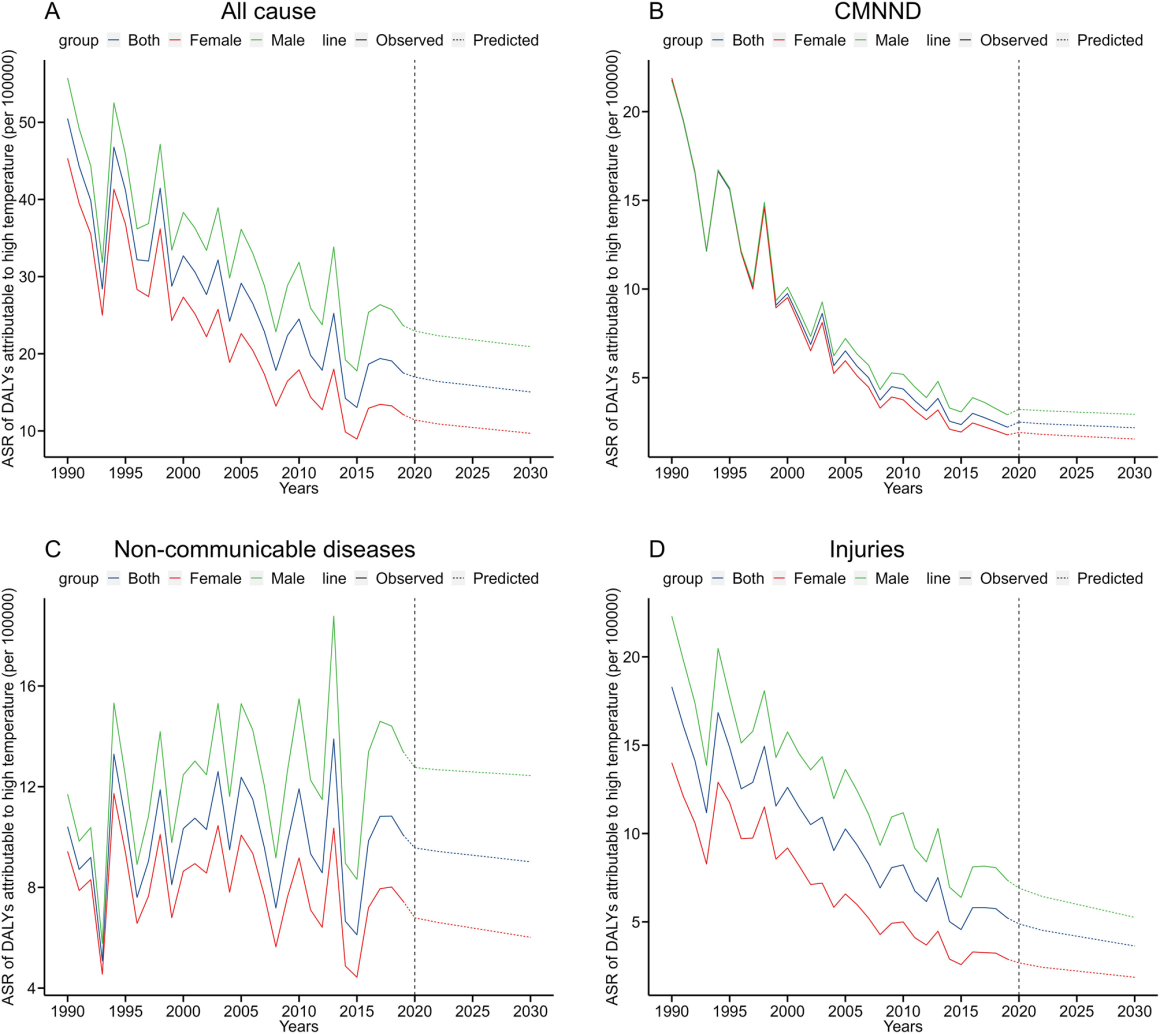
Prediction of DALYs due to high temperatures in Mainland China from 2020 to 2030. (**A**) All cause; (**B**) CMNND; (**C**) non-communicable diseases; (**D**) injuries. *CMNND* communicable, maternal, neonatal, and nutritional diseases.

Table 2 predicts the disease burden due to high temperatures in Mainland China from 2020 to 2030 using the R software. The predicted ASR of deaths due to high temperatures in 2030 is 0.82/100,000, with 1.13/100,000 for males and 0.57/100,000 for females. The highest contribution to the ASR of deaths due to high temperatures came from non-communicable diseases (0.61/100,000), followed by CMNND (0.11/100,000) and injuries (0.07/100,000). As shown in Fig. 4A–D, the predicted trend of the ASR of deaths due to high temperatures from 2020 to 2030 showed a significant decrease in CMNND and injuries, while the ASR of deaths due to overall cause and non-communicable diseases remained relatively stable. The predicted ASR of DALYs attributed to high temperatures in 2030 is 15.05/100,000, with 20.92/100,000 for males and 9.69/100,000 for females. Similarly, the largest contributing ASR of DALYs due to high temperatures comes from non-communicable diseases (9.02/100,000), followed by injuries (3.63/100,000) and CMNND (2.18/100,000). As shown in Fig. 5A–D, the predicted ASR of DALYs due to high temperatures from 2020 to 2030 among the overall cases, as well as CMNND and injuries, showed an overall decreasing trend, while the non-communicable disease group showed a stable trend.

**Table 2:** Prediction of disease burden due to high temperature in Chinese Mainland from 2020 to 2030.

Years	ASR of deaths (per 100,000)			ASR of DALYs (per 100,000)		
	Both	Male	Female	Both	Male	Female
All cause						
2020	0.89	1.21	0.64	16.98	22.95	11.42
2021	0.88	1.20	0.63	16.69	22.65	11.16
2022	0.87	1.19	0.62	16.40	22.35	10.90
2023	0.86	1.18	0.61	16.24	22.17	10.75
2024	0.86	1.18	0.61	16.07	21.99	10.60
2025	0.85	1.17	0.60	15.91	21.81	10.45
2026	0.84	1.16	0.59	15.74	21.63	10.30
2027	0.84	1.15	0.59	15.57	21.45	10.14
2028	0.83	1.15	0.58	15.39	21.28	9.99
2029	0.82	1.14	0.58	15.22	21.10	9.84
2030	0.82	1.13	0.57	15.05	20.92	9.69
CMNND						
2020	0.13	0.19	0.09	2.50	3.22	1.92
2021	0.13	0.19	0.09	2.45	3.18	1.86
2022	0.12	0.19	0.08	2.40	3.14	1.81
2023	0.12	0.18	0.08	2.38	3.12	1.77
2024	0.12	0.18	0.08	2.35	3.09	1.74
2025	0.12	0.18	0.08	2.32	3.06	1.71
2026	0.12	0.18	0.08	2.29	3.04	1.67
2027	0.12	0.18	0.08	2.26	3.01	1.64
2028	0.11	0.17	0.07	2.24	2.99	1.61
2029	0.11	0.17	0.07	2.21	2.96	1.58
2030	0.11	0.17	0.07	2.18	2.93	1.54
Non-communicable diseases						
2020	0.66	0.87	0.49	9.57	12.76	6.79
2021	0.65	0.87	0.49	9.50	12.72	6.70
2022	0.65	0.86	0.48	9.43	12.67	6.61
2023	0.64	0.86	0.47	9.38	12.64	6.53
2024	0.64	0.85	0.47	9.33	12.61	6.46
2025	0.63	0.85	0.46	9.28	12.59	6.39
2026	0.63	0.84	0.46	9.23	12.56	6.31
2027	0.62	0.84	0.45	9.17	12.53	6.24
2028	0.62	0.83	0.45	9.12	12.50	6.16
2029	0.61	0.83	0.44	9.07	12.47	6.09
2030	0.61	0.83	0.44	9.02	12.44	6.02
Injuries						
2020	0.09	0.13	0.06	4.89	6.91	2.68
2021	0.09	0.13	0.05	4.70	6.67	2.55
2022	0.09	0.12	0.05	4.52	6.44	2.43
2023	0.08	0.12	0.05	4.41	6.29	2.36
2024	0.08	0.12	0.05	4.30	6.14	2.29
2025	0.08	0.11	0.05	4.19	6.00	2.22
2026	0.08	0.11	0.04	4.08	5.85	2.15
2027	0.08	0.11	0.04	3.97	5.70	2.08
2028	0.07	0.10	0.04	3.86	5.55	2.01
2029	0.07	0.10	0.04	3.74	5.40	1.93
2030	0.07	0.10	0.04	3.63	5.26	1.86

*CMNND* communicable, maternal, neonatal, and nutritional diseases.

## Discussion

This study analyzes the trends of disease burden due to high temperatures in Mainland China from 1990 to 2019 using data from GBD 2019 and makes a prediction for the next 10 years. These results suggest that the disease burden due to high temperatures in Mainland China remains heavy. The number of deaths showed a further upward trend, but it is gratifying that the ASR of deaths and DALYs showed a downward trend. In addition, the etiology due to high temperatures changed between 1990 and 2019, with the main contribution coming from CMNND in 1990 and transitioning to non-communicable diseases in 2019. The ASR of deaths and DALYs due to high temperatures in males were higher than those in females, while the ASR of deaths and DALYs due to high temperatures increased with age. Predictive analysis showed that the ASR of deaths and DALYs due to high temperatures will continue to decline from 2020 to 2030, whereas that of non-communicable diseases due to high temperatures will remain stable for the next 10 years. This study provides a comprehensive understanding of the impact of high temperatures on health under global warming trends, which will be helpful for formulating public health policies.

The disease burden due to high temperatures in Mainland China shows significant fluctuations, which are related to the temperature fluctuations in China^19^. A study by Li et al. showed that the national average temperature showed a significant upward trend from 1964 to 2008^20^. Lan et al. analyzed data from 118 meteorological stations in China from 1969 to 2018 and showed a significant trend of warming in all seasons, with rates of increase in spring, summer, autumn, and winter at 0.347, 0.125, 0.200, and 0.302 °C/10a, respectively^21^. This finding can be attributed to changes in work patterns. With the continuous advancement of industrialization and urbanization, manual labor, which must withstand high temperatures, is gradually being replaced with machines and industrial automation. For example, traditional manual labor, such as farming, steelmaking, and mining, has begun to be mechanically automated, whereas positions such as technical personnel, administrative personnel, financial practitioners, and sales personnel are currently increasing and are mainly concentrated in office environments^22^. In addition, improvements in hygiene conditions are also an important reason for the decreased burden due to high temperatures. In the past, poor hygiene conditions in China led to an outbreak of infectious diseases in a large population, seriously threatening human life and health. With continuous progress in medical technology and significant improvements in living conditions, the incidence rates have gradually declined and cure rates have greatly improved of many infectious diseases^23^. However, with the increasing use of air conditioning in offices owing to global warming, carbon emissions have greatly increased, accelerating the progress of global warming and creating a vicious cycle where humans will have nowhere to hide, which requires the attention and participation of all humanity.

The ASR of deaths and DALYs caused by non-communicable diseases due to high temperatures is still relatively high, and we should attach great importance to them.

There is a certain relationship between global warming and the increase in non-communicable diseases^24^. Firstly, global warming has exacerbated the problem of air pollution, especially particulate matter and ozone in the atmosphere. These pollutants are closely related to the risk of non-communicable diseases such as heart disease, stroke, respiratory diseases, and cancer^25^. Secondly, global warming exacerbates extreme weather events such as heatwaves, droughts, floods, and hurricanes. These weather events can lead to heat stress, dehydration, insufficient food and water supply, thereby increasing the risk of diseases such as heart disease, respiratory system disease, kidney disease, and stroke^26,27^. Finally, global warming has a complex impact mechanism on the increase of non-communicable diseases. Therefore, comprehensive measures need to be taken to address climate change, including reducing greenhouse gas emissions, improving air quality, improving health education, and responding to extreme weather events^28,29^. These measures help reduce the risk of non-communicable diseases and promote human health and well-being.

The ASR of deaths and DALYs due to high temperatures in males were higher than those in females. There are indeed studies indicating gender differences in high-temperature tolerance^30^, but the specifics have not yet been determined. Some studies suggest that there are differences in physiological structure and function between males and females. For example, men usually have larger bodies than women, relatively smaller surface area, and weaker heat dissipation ability^31^. In certain cultural and social environments, men may be more inclined to exhibit adventurous and courageous behavior, leading to them not paying attention to health risks in high-temperature environments. On the contrary, women may be more inclined to adopt conservative behavior and prioritize health and safety^32^.

The aging population is another reason for the rising number of deaths due to high temperatures, and our study also confirms that the disease burden due to high temperatures is directly proportional to age, which is consistent with other research results^33^. The aging population in China has been accelerating since 1990, and by 2020, the population aged 65 years and above has reached 190 million^34^. Research by Yang et al. showed that the aging population increased the number of deaths due to high temperature by 2.3–5.8 times. Data from South Korea also show that the mortality rate related to high temperatures in South Korea increased by 4–6 times from 1992 to 2010. After removing the impact of population aging, the mortality rate only increased by 0.5 to 1.5 times^35^. Therefore, with the deepening of misconduct in the aging population, it is of great significance to formulate relevant policies to reduce high-temperature exposure in the elderly and help them improve their self-protection and prevention awareness.

This study had certain limitations. First, the scope of this study mainly covered very large countries. Owing to the large land area of China and significant temperature changes in various regions, the large number of research units may cause difficulties in the implementation of specific health policies. Second, the data of this study were from the GBD database in 2019, and the indicators related to the burden of disease were converted from different models; thus, there is a high requirement for the authenticity of the data. Although GBD uses standardized tools and processes to improve the accuracy and robustness of data, there are still differences and biases in the data transmission process.

## Conclusions

In summary, this study revealed the disease burden caused by high temperatures in Mainland China from 1990 to 2019 and predicted the burden from 2020 to 2030. The results indicate that the disease burden due to high temperatures in Mainland China is still relatively heavy and is mainly related to population aging and an increase in non-communicable diseases.

## Data Availability

The validation dataset was available on request. Data were obtained from the 2019 GBD of Disease database (https://vizhub.healthdata.org/gbd-results/). The GBD dataset was hosted by the Institute for Health Metrics and Evaluation at Washington University.

## Abbreviations

GBD: Global Burden of Disease
DALYs: Disability adjusted life years
CMNND: Communicable, maternal, neonatal, and nutritional diseases
ASR: Age standardized rate
EAPC: Estimated annual percentage change
